# Playgroup Participation and Social Support Outcomes for Mothers of Young Children: A Longitudinal Cohort Study

**DOI:** 10.1371/journal.pone.0133007

**Published:** 2015-07-16

**Authors:** Kirsten J. Hancock, Nadia K. Cunningham, David Lawrence, David Zarb, Stephen R. Zubrick

**Affiliations:** 1 Telethon Kids Institute, The University of Western Australia, Perth, Western Australia, Australia; 2 Playgroup WA (Inc), North Perth, Western Australia, Australia; University of São Paulo, BRAZIL

## Abstract

**Objective:**

This study aimed to examine friendship networks and social support outcomes for mothers according to patterns of playgroup participation.

**Methods:**

Data from the Longitudinal Study of Australian Children were used to examine the extent to which patterns of playgroup participation across the ages of 3–19 months (Wave 1) and 2–3 years (Wave 2) were associated with social support outcomes for mothers at Wave 3 (4–5 years) and four years later at Wave 5 (8–9 years). Analyses were adjusted for initial friendship attachments at Wave 1 and other socio-demographic characteristics.

**Results:**

Log-binomial regression models estimating relative risks showed that mothers who never participated in a playgroup, or who participated at either Wave 1 or Wave 2 only, were 1.7 and 1.8 times as likely to report having no support from friends when the child was 4–5 years, and 2.0 times as likely to have no support at age 8–9 years, compared with mothers who persistently participated in playgroup at both Wave 1 and Wave 2.

**Conclusion:**

These results provide evidence that persistent playgroup participation may acts as a protective factor against poor social support outcomes. Socially isolated parents may find playgroups a useful resource to build their social support networks.

## Introduction

Social support is widely understood to have positive effects on health and wellbeing [1–4]. Although there is some debate surrounding how it can best be defined [1], social support comprises at least three types of support; tangible or instrumental support (the provision of aid or services), informational support (the provision of information or advice) and emotional support (being able to confide in and rely on others; [5]). At its core, social support is based upon personal relationships in which people believe they are cared for and valued, and belong to a network of communication and mutual obligation [6]. Numerous studies have demonstrated associations between social connectedness and engagement and both physical and psychological wellbeing [7,8].

Social support is particularly recognised as an important resource for parents of young children. The transition to parenthood can be a challenging period for many parents, of enduring stress, financial adjustments, upheaval of life, new responsibility and sleep deprivation. In addition, loneliness or social isolation may occur [9]. In this context, the availability of social support may help to buffer against the effects of stress [8]. The support may be delivered in terms of informal child care or financial support by relatives (i.e. instrumental support), advice about parenting practice (i.e. informational support) or through social ties and interpersonal relationships (i.e. emotional support) [10]. As in the broader social support literature, the benefits of social support for parents in particular are well recognised. For example, higher levels of social support have been linked with better health for women pre- and post-natally [11], lower rates of depression and stress [12,13], increased parent self-efficacy [13,14], and more secure mother-infant attachments [15].

Despite knowledge about the value of social support for new parents, and mothers in particular, Parry et al. [16] suggested that the transition into motherhood is more socially isolating in modern times than in the past, with factors such as increased workforce participation, increased geographic mobility and distance from family members, and higher levels of lone parenthood resulting in more sole parents parenting with less social support. These societal changes, in conjunction with the value of social support, mean that programs or services that promote the development of social networks for parents, particularly parents who are socially isolated, can therefore be a valuable tool for new parents.

A range of opportunities to expand social networks may be available to parents of young children, including child care or early learning centres, preschools, community events, mothers groups and playgroups. Research from the United States has shown that enrolling children in child care centres may lead to the development of new friendships and extended networks, compared with mothers who do not use child care services, particularly if those centres promote socialisation among parents [17]. However, as child care participation is strongly linked with labour force status and affordability [18], not all parents can (or need to) access formal child care. Australian estimates of formal child care participation suggest around 10% of 0–1 year olds and 40% of 2–3 year olds attended formal care of varying duration, and most were attending so that caregivers could meet their work or study commitments [19]. Though some Australian parents will likely form social support networks through child care participation, as found in the US, a large proportion do not have this opportunity.

In Australia, an alternative, targeted opportunity for parents of young children to build their social networks is playgroup participation. Playgroups, a common way for new parents to engage with others, are regular gatherings of parents (or other caregivers) and children under the age of five. Though formats can vary, they are typically held once a week for around 2 hours in a variety of locations, including the homes of participants, in schools or community halls, or parks and playgrounds. Importantly, playgroups are distinct from child care or crèche arrangements, as caregivers stay for the duration of the playgroup and participate in activities with their children and socialise with other caregivers.

Two broad playgroup models operate in Australia, community and supported playgroups. Community playgroups, which are parent-led and managed by participants, are the most common. Supported playgroups are developed and supported by State and Territory playgroup associations and other not-for-profit agencies, where a facilitator is employed to organise the activities undertaken at the playgroup. These playgroups are supported by funding from both federal and state government bodies, and are often offered to disadvantaged communities where the development and management of playgroups can be difficult. These playgroups are supported in recognition of the broad objectives that playgroups aim to achieve: to improve the wellbeing of parents and children, to improve parenting skills and family functioning, and to develop stronger communities.

Evidence regarding the extent to which playgroups meet these objectives is relatively scant, despite the large proportion of Australian families that access them. Prior research using data from the Longitudinal Study of Australian Children showed over 60% of the study children born in 2004–2005 had accessed a playgroup at least once by the age of 4–5 years [20]. This study also showed that children from disadvantaged families performed significantly better on measures of learning competency and social and emotional wellbeing if they persistently attended playgroup across the ages of 0 to 3 years than disadvantaged children who never attended a playgroup. Yet, disadvantaged families were significantly less likely to participate in playgroups than their non-disadvantaged counterparts. In an Australian qualitative study, Strange et al. [21] found that mothers of young children in newer residential areas reported that through playgroup attendance they were able to form friendships, build a supportive network, and had an increased sense of community connectedness.

Other studies have shown that parent group experiences are not always positive for those who participate. In a Canadian qualitative study, for example, Mulcahy et al. [22] found that participating in an informal mothers group enabled some mothers to ‘get together, get by and get ahead’ whilst others ‘get left out, get judged and get gendered’. Some mothers reported excluding some participants because of differing attitudes towards parenting. Thus, where differences exist amongst members of the group, the experience may be less positive overall. This is in line with research showing that mothers tend to seek support from others of similar backgrounds [14]. While playgroups are more structured and organised than informal mothers groups, and can provide access to other services including conflict mediation that may help to ameliorate relationship issues, it is likely that the social benefits of playgroups are influenced by both individual factors (e.g., demographics and personality traits such as sociability, extraversion, and social competence) and group dynamics, as is the case with other social networks [23].

The aim of this study was to examine the extent to which playgroup participation relates to social support outcomes for Australian mothers, and to support received from friends in particular. This study expands on the previous research of Hancock et al. [20], using data collected for the Longitudinal Study of Australian Children (LSAC). We examined the likelihood of mothers having no support from friends when their child was aged 4–5 years according to the child’s participation in playgroup at age 3–19 months and 2–3 years. We also assessed the same friendship support outcome for mothers when their child was 8–9 years to examine the extent to which the friendship supports endured longer-term. Given the previous research demonstrating poorer friendship outcomes for mothers whose children did not attend child care [17]—organisations that are arguably less accessible and less focussed on promoting social networks than playgroups—we hypothesised that mothers who do not participate in playgroups will have a greater risk of having no support from friends, relative to mothers who do participate, both in the short-term and long-term, after controlling for initial levels of support reported by mothers when their child was aged 3–19 months and other socio-demographic factors.

## Methods

### Participants

This study used data collected from LSAC, a nationally representative study of Australian parents and their children over time. Two cohorts of children (B and K) were recruited into the study: the B cohort consisted of 5,107 infants aged 3–19 months, and the K cohort consisted of 4,983 children aged 4–5 years. The first wave of data collection took place in 2004, and children were followed up every two years, with Wave 5 data collected in 2012. This study used B cohort data from Wave 1 (3–19 months) and Wave 2 (2–3 years) to assess patterns of playgroup participation, and Wave 3 (4–5 years) and Wave 5 (8–9 years) to examine friendship support outcomes. The sampling methodology and design of LSAC are extensively detailed elsewhere (see Soloff, Lawrence, & Johnstone [24]; Soloff, Lawrence, Misson & Johnstone [25]). Briefly, the LSAC used a two-stage clustered sample design, with Australian postcode areas as the primary sampling unit. Approximately one-in-ten Australian postcode areas were randomly selected and children were then randomly selected within postcodes using the Medicare enrolment database as the sampling frame, ensuring that only one child per household was selected. The Medicare database had good coverage, with more than 90% of infants estimated to be enrolled on the database by 4 months of age [24]. The response rate for the B cohort at Wave 1 was 53.6%. The B cohort sample consisted of 4,606 children aged 2–3 years at Wave 2 (90.2% response rate), 4,386 children aged 4–5 years at Wave 3 (85.9% of Wave 1 sample) and 4,085 children aged 8–9 years at Wave 5 (80.0% of Wave 1 sample). Design, sample and population weights were calculated at each wave to ensure adequate representativeness of the data and to account for bias in sample attrition [25–27].

### Ethics Statement

The Longitudinal Study of Australian Children (LSAC) is conducted in a partnership between the Department of Social Services, the Australian Institute of Family Studies and the Australian Bureau of Statistics. The study has ethics approval from the Australian Institute of Family Studies Ethics Committee. The Ethics Committee is registered with the Australian Health Ethics Committee, a subcommittee of the National Health and Medical Research Council (NHMRC). As the study children were all minors at the time these data were collected, written informed consent was obtained from the caregiver on behalf of each of the study children. The signed consent forms are retained by the field agency.

### Data Collection

Data were collected at each wave from multiple informants, using a variety of methods. The primary caregiver of the study child (Parent 1) was the main provider of information, who in most cases was the biological mother of the study child (98.3% at Wave 1, 97.9% at Wave 2 and 97.6% at Wave 3). Parent 1 was asked to complete an in-home interview as well as a self-complete questionnaire at each wave. At Wave 2, the questionnaire was divided into two surveys, one to be completed during the home visit (in-home survey), and the other to be completed and returned at a later time (leave-behind survey). Parent 1 response rates on the self-complete questionnaires were 85% at Wave 1, 98% for the in-home survey and 76.8% for the leave-behind survey at Wave 2, and 87.4% at Wave 3. At Wave 5 the self-complete questionnaires were replaced with computer-assisted self-interviews that were completed during the home visit with a 98% response rate. Questionnaires were also completed by Parent 2, the study child themselves, parents living elsewhere, teachers and child care workers where appropriate.

As this study was concerned with support outcomes for mothers, and because the vast majority of primary caregivers in the study were mothers of the study children, analyses were restricted to cases where social support information was provided by mothers.

### Measures

#### Support from friends

The key outcome measure was support received from friends, which was a single-item measure collected at Wave 3 (4–5 years) and Wave 5 (8–9 years). Mothers were asked in the self-complete surveys “how often do you receive support from friends in raising study child?” Responses included ‘always’, ‘often’, ‘sometimes’, ‘rarely’ or ‘never’, with an additional option for ‘don’t have friends’. To address the hypothesis that mothers who do not participate in playgroup are at greater risk of having no support from friends, we identified mothers as having no support if they had responded that they never received support from friends, or did not have friends. All other responses were coded as having at least some support.

The selected measure addresses the frequency of support received from friends, however it does not capture the type of support (i.e. instrumental, informational or emotional), the strength of attachment, or whether mothers actually need any social support from friends. For example, one mother may frequently receive low-level support (e.g. day to day parenting advice from other mothers), where another may only need occasional, but more invested support, such as emergency child care. Other assessments used in the literature typically capture different types of support, for example the Medical Outcomes Study (MOS) Social Support Survey [28], but they do not determine the source of social support. Irrespective of support needs, support type or quality of support provided, mothers who indicate that they do not have friends or who say they never receive support will not receive *any* type of support from friends. To the extent that regular playgroup participation would be associated with the *availability* of friendship supports—a necessary, though not sufficient requisite for getting support—the selected measure was considered appropriate for examining the relationship between playgroup participation and the availability of social support, or lack thereof, from friends.

As the measure of support from friends consisted of only one item, the measure was compared to other broad indicators of friendship and support, including the frequency of contact with friends, and how often mothers felt they needed support but couldn’t get it. When asked, “How often do you see, talk to or email your friends?” at both Wave 3 and 5 a substantially lower proportion of mothers with no support from friends said they were in contact with friends at least once a week compared with mothers with some support (Wave 3, 43% v. 80%, *p* < .001; Wave 5, 48% v. 78%, *p* < .001). Similarly, a higher proportion of mothers with no support said they were in contact with friends fewer than a few times year (Wave 3, 28% v. 3%, *p* < .001; Wave 5, 23% v. 5%, *p* < .001). Regarding perceived support needs, a higher proportion of mothers with no support from friends reported they often or very often needed support but couldn’t get it from anyone compared with mothers with at least some support (Wave 3, 22% v. 10%, *p* < .001; Wave 5, 12% v. 8%, *p* = .005).

#### Friendship networks

It is possible that mothers who did not have friendship networks around the time the study child was born may also have lacked the social confidence, awareness or motivation that might otherwise encourage them to participate in a playgroup. As such, any association between playgroup participation and social support outcomes in later years may simply reflect a situation where mothers do not participate in playgroups because they didn’t have the initial support or confidence. Therefore we assessed indicators of friendship and support reported by mothers at Wave 1 (3–19 months). At Wave 1 the survey items relating to social support did not differentiate whether the support was provided by family or friends. However, three items assessing attachment to friends were included. While having friendship attachments does not mean that social support is necessarily provided by those friendships, a lack of friends or friendship attachments likely precludes receiving social support from friends. Therefore the attachment to friends measure was used as a proxy of social support from friends at Wave 1.

The attachment to friends measure was included in the Wave 1 leave-behind questionnaire and consisted of 3 items: ‘I feel closely attached to my friends’, ‘my friends take notice of my opinions’, and ‘sometimes I feel excluded among my friends’. Responses ranged from 1 = totally agree to 5 = totally disagree, with a further option for “no friends”. Responses were summed, with the third item concerning exclusion being reverse coded. Mothers with a total score of 12 or above—meaning they disagreed with all three items, or if they indicated they did not have friends—were coded as having poor friendship attachment. All other mothers were coded as having at least some attachment to friends.

As the measure of social support at Wave 1 relates more to the absence of social support than its presence, the attachment to friends measure was compared to other broader social support indicators to assess its validity as a proxy measure. Compared with mothers with adequate friendship attachments, at Wave 1 mothers with poor attachment were more likely to report that they did not receive enough support from family and friends at (37% v. 20%, *p* < .001), and that they often or very often needed support but could not get it (21% v. 7%, *p* < .001). Mothers with poor attachment to friends were also less likely than mothers with adequate attachment to nominate friends as a top 3 source of parenting information (46% v. 66%, *p* < .001), practical help (45% v. 63%, *p* < .001), emotional support (63% v. 86%, *p* < .001) and financial help (16% v. 21%, *p* = .019).

#### Playgroup participation

At Waves 1, 2 and 3 Parent 1 was asked “In the past 12 months, have you used any of the following services for the study child… Playgroups or parent-child groups?” and could respond either ‘yes’ or ‘no’. The items were collected from the self-complete survey at Wave 1, the self-complete leave-behind survey at Wave 2, and the face-to-face interview at Wave 3. As there were no further questions on the type of playgroup attended, or the frequency of participation, we cannot distinguish between children who attended playgroup consistently throughout the previous 12 months and those who attended infrequently. We also could not ascertain who participated in playgroups with the study child, but assume that the majority of children attending playgroup did so with their mother. We also could not distinguish between the types of playgroups that families were attending.

Previous research using the same data set showed that 40% of respondents indicated the study child had used a playgroup at age 0–1 year (Wave 1), 53% had participated at age 2–3 years (Wave 2) and 25% at age 4–5 years (Wave 3) [20]. For consistency with the previously published work our measure of playgroup participation excluded participation at Wave 3, as the study children were moving into more formal education settings by age 4. Thus, this study focused on the pattern of playgroup participation at ages 3–19 months (Wave 1) and 2–3 years (Wave 2).

#### Socio-demographic covariates

Data on a range of covariates were also collected via parent report, including: mother’s age at birth of first child, mother’s employment status, mother’s highest level of education, family structure, language spoken in the home, household income and maternal mental health. At Wave 1, household income was collected by asking respondents to select from a category (i.e. range of income). These categories were then broadly grouped into quartiles. At subsequent waves more detailed information was collected allowing the calculation of equivalised household income, adjusting for the number of people in the household. Maternal mental health was assessed using the Kessler 6 scale (K6 [28]). Scores of 13 and above indicate a probable serious mental illness [29]. As the LSAC is a non-clinical sample, very few mothers fell above this cut-point. In concert with other studies [30,31] we used a lower cut-point of 8+ to classify mothers as having elevated non-specific psychological distress.

When considering the families that participated not only in all relevant interviews, but also completed and returned all of the self-complete questionnaires, and where respondents were mothers of the study child, the final sample size consisted of 2,617 mothers at Wave 3 (4–5 years) and 2,576 at Wave 5 (8–9 years). To assess potential bias due to attrition and non-response, the Wave 1 (3–19 months) characteristics of the families contributing to the Wave 3 and Wave 5 analyses were compared with the characteristics of the full Wave 1 sample (Table 1). The analytic samples at Wave 3 and Wave 5 were slightly overrepresented in terms of labour force participation, being older first-time mothers, having higher household incomes, two-parent families, and speaking English in the home. Minor differences in distributions were observed for Wave 1 attachment to friends, needing support, mother’s highest education mother’s highest education, and mother’s mental health.

**Table 1:** Wave 1 characteristics of the full Wave 1 sample (3–19 months), and the analytic samples at Wave 3 (4–5 years) and Wave 5 (8–9 years).

	Full Wave 1 Sample	Wave 3 Analytic Cases	Wave 5 Analytic Cases
Characteristic	%	%	%
**Attachment to friends (N)**	**4,215**	**2,617**	**2,576**
Poor attachment	18.9	16.7	16.3
Adequate attachment	81.1	83.3	83.7
**Needed but couldn’t get support (N)**	**3,768**	**2,617**	**2,576**
Often/Very often	10.0	8.7	8.6
Sometimes	46.1	46.5	45.6
Rarely	43.8	44.9	45.7
**Mother’s highest education (N)**	**4,960**	**2,615**	**2,574**
Year 10 or less	15.1	8.8	9.0
Year 11 or 12	18.4	17.0	17.1
Diploma or certificate	37.1	37.3	36.3
Bachelor degree or higher	29.5	37.0	37.6
**Mother’s employment (N)**	**4,960**	**2,610**	**2,568**
Full-time	10.7	10.7	10.8
Part-time	28.3	32.2	32.2
Employed on maternity leave	9.0	11.7	12.1
Seeking work	3.4	2.4	2.2
Not in labour force	48.7	43.0	42.7
**Mothers age at birth of first child (N)**	**4,960**	**2,615**	**2,573**
Less than 20 years	10.6	4.3	4.0
20–24 years	22.5	16.0	16.2
25 years +	67.0	79.7	79.8
**Household income (N)**	**4,705**	**2,503**	**2,466**
Less than $700 per week	25.8	17.5	17.3
$700-$999 per week	21.6	21.2	21.0
$1000–1499 per week	26.0	30.0	30.0
$1500 or more per week	26.5	31.3	31.7
**Family structure (N)**	**4,972**	**2,616**	**2,575**
Single parent family	10.2	6.1	5.5
Two-parent or other family	89.8	93.9	94.5
**Language spoken in home (N)**	**4,974**	**2,617**	**2,576**
English	87.3	91.9	91.6
Other	12.7	8.1	8.4
**Mother’s mental health (N)**	**4,170**	**2,588**	**2,550**
Within normal range	87.2	89.0	88.9
Elevated psychological distress	12.8	11.0	11.1

### Statistical Analysis

Firstly, the proportion of mothers with no support from friends at Waves 3 (4–5 years) and 5 (8–9 years) was cross-tabulated with the playgroup participation variable and other covariates to explore the distribution patterns and to determine which covariates would then be included in the multivariate analysis.

Next, we used multivariate log-binomial regression to generate the adjusted relative risk of mothers having no support from friends according to playgroup attendance pattern. Separate models were used to estimate the adjusted relative risk of having no support at Wave 3 (4–5 years) and Wave 5 (8–9 years). In addition to playgroup attendance, Wave 1 attachment to friends and the frequency of needing but not getting support were included as controls for initial measures of friendship networks and general support. All covariates that were significantly associated with Wave 3 or Wave 5 support outcomes in the exploratory analysis were included as covariates in the regression analyses. Results are reported as risk ratios (RR) and 95% confidence intervals. The adjusted probabilities of having no support from friends for each level of playgroup participation were also estimated.

SAS 9.4 [32] was the statistical software package used for all analyses, and longitudinal weights were used to account for sample attrition bias across waves. The log-binomial regressions were estimated using the GENMOD procedure. Correlation among families living in the same postcode was accounted for using the ‘repeated’ statement.

## Results

Table 2 displays the proportions of mothers reporting no support from friends at Wave 3 (4–5 years) and Wave 5 (8–9 years) according to the pattern of playgroup participation, as well as the demographic characteristics of the sample. In total, 12.7% of mothers reported having no support from friends at Wave 3, and 14.5% at Wave 5. For mothers who reported poor attachment to friends at Wave 1, these proportions were significantly higher (24.3% at Wave 3; 26.8% at Wave 5), and likewise for mothers who had reported they often needed support but couldn’t get it (23.4% and 24.0% respectively).

**Table 2:** Proportion of mothers with no support from friends at Wave 3 (4–5 years) and Wave 5 (8–9 years), by playgroup participation and selected family and demographic characteristics.

	Wave 3 (4–5 years)			Wave 5 (8–9 years)		
Characteristic	N	% (SE)	Chi-Square Test	*N*	% (SE)	Chi-Square Test
**All mothers**	2,617	12.7 (0.7)		2,541	14.5 (0.8)	
**Attachment to friends** ^a^						
Poor	416	24.3 (2.1)	χ^2^(1) = 58.7, *p* < .001	394	26.8 (2.2)	χ^2^(1) = 58.1, *p* < .001
Adequate	2,201	10.3 (0.6)		2,147	12.0 (0.7)	
**Frequency of needing support and not getting it** ^a^						
Often/Very often	217	23.4 (3.0)	χ^2^(2) = 23.1, *p* < .001	210	24.0 (3.1)	χ^2^(2) = 15.9, *p* < .001
Sometimes	1,203	12.5 (0.9)		1,158	15.0 (1.1)	
Rarely	1,197	10.8 (1.0)		1,173	12.2 (1.0)	
**Playgroup participation** ^b^						
None	825	14.9 (1.1)	χ^2^(2) = 20.7, *p* < .001	798	18.0 (1.5)	χ^2^(2) = 26.0, *p* < .001
Either Wave 1 or 2	918	14.7 (1.2)		879	16.7 (1.4)	
Both Wave 1 and 2	874	8.1 (0.9)		864	8.5 (1.0)	
**Mother’s highest education** ^c^						
Year 10 or less	151	15.2 (3.1)	χ^2^(3) = 8.5, *p* = .036	123	27.7 (4.2)	χ^2^(3) = 30.1, *p* < .001
Year 11 or 12	389	14.6 (1.6)		296	11.6 (1.8)	
Diploma or certificate	988	14.0 (1.1)		987	16.2 (1.3)	
Bachelor degree or higher	1,088	10.0 (0.9)		1,128	10.8 (1.0)	
**Mother’s employment** ^c^						
Full-time	522	15.6 (1.6)	χ^2^(3) = 12.8, *p* = .005	823	14.1 (1.3)	χ^2^(3) = 13.1, *p* = .004
Part-time	1,195	10.0 (0.8)		1,149	12.2 (1.1)	
Maternity leave	57	18.9 (5.9)		d	-	
Not employed or in labour force	843	14.1 (1.2)		557	19.1 (1.8)	
**Mother’s age at birth of first child** ^a^						
Less than 20 years	102	14.1 (3.6)	χ^2^(2) = 0.19, *p* = .908	89	19.5 (4.7)	χ^2^(2) = 1.6, *p* = .448
20–24 years	417	12.7 (1.8)		400	14.3 (1.9)	
25 years +	2,096	12.5 (0.7)		2,050	14.2 (0.8)	
**Equivalised household income tertile** ^c^						
Lowest	693	15.3 (1.4)	χ^2^(2) = 7.6, *p* = .023	629	17.9 (1.6)	χ^2^(2) = 12.3, *p* = .002
Middle	849	10.2 (1.1)		791	11.8 (1.9)	
Highest	880	12.3 (1.1)		816	11.7 (1.2)	
**Family structure** ^c^						
Single parent family	216	19.1 (3.1)	χ^2^(1) = 6.1, *p* = .014	285	14.6 (2.2)	χ^2^(1) = 0.02, *p* = .967
Two-parent family	2,397	12.0 (0.6)		2,255	14.5 (0.8)	
**Language spoken in home** ^c^						
English	2,441	11.9 (0.7)	χ^2^(2) = 12.0, *p* < .001	2,369	12.9 (0.7)	χ^2^(1) = 32.2, *p* < .001
Other	176	21.0 (2.9)		172	29.4 (3.4)	
**Mother’s mental health** ^c^						
Within normal range	2,354	12.0 (0.7)	χ^2^(1) = 10.4, *p* = .001	2,328	14.1 (0.8)	χ^2^(1) = 1.5, *p* = .216
Elevated psychological distress	234	20.1 (2.6)		212	18.0 (3.1)	

^a^ Measured at Wave 1

^b^ Measured across Wave 1 and Wave 2

^c^ Measures taken from the concurrent Wave (3 or 5).

^d^ Excluded for small cell size at Wave 5.

The descriptive analyses showed that a significantly higher proportion of mothers had no support from friends if they never participated in playgroup compared with mothers who persistently attended when their child was aged 3–19 months and 2–3 years (Wave 3 = 14.9% v. 8.1%; Wave 5 = 18.0% v. 8.5%). For the socio-demographic variables, a higher proportion of mothers reported having no support where they had lower levels of education, if they were not employed part-time, if they had lower levels of income, were single parents (significant at Wave 3 only), if they did not speak English in the home, or had likely elevated psychological distress (significant at Wave 3 only, see Table 2).

Two log-binomial regression models (Table 3) were then fitted to further examine the association between playgroup participation and the risk ratios of having no support from friends when their child was aged 4–5 years (Wave 3) and 8–9 years (Wave 5). After adjusting for initial friendship networks, social support needs and socio-demographic characteristics, the relative risk of having no support from friends at Wave 3 (4–5 years) was 1.7 times higher for mothers who never participated in playgroup, and 1.8 times higher for mothers who participated at either Wave 1 (3–19 months) or Wave 2 (2–3 years), relative to mothers who persistently participated at both Wave 1 and Wave 2. The adjusted proportions of mothers having no support from friends at Wave 3 was 12.8% for those who never participated, 13.6% for those who participated at one wave only, and 7.7% for those who participated in playgroup at both waves. At Wave 5, approximately four years later, the relative risks of having no support from friends at Wave 5 (8–9 years) were 2.0 times higher for both mothers who either never participated in playgroup and those who participated at only Wave 1 or 2. The adjusted proportions of mothers having no support from friends at Wave 5 was 14.7% for those who never participated, 14.4% for those who participated at one wave only, and 7.3% for those who participated in playgroup at both waves. Post-hoc analyses using the ESTIMATE statement indicated there was no difference in the relative risk of having no support from friends at either Wave 3 or Wave 5 when comparing mothers who never participated in playgroup with those who participated at one wave only (Wave 3: RR = 1.03, 95% CI 0.8–1.3, *p* = .803; Wave 5: RR = 1.02, 95% CI 0.8–1.4, *p* = .876).

**Table 3:** Results of log-binomial regression models estimating adjusted relative risk (RR) of having no support from friends, Wave 3 (4–5 years, *n* = 2,392) and Wave 5 (8–9 years, *n* = 2,351).

	Wave 3 (4–5 years)			Wave 5 (8–9 years)		
Variable	RR	95% CI	p-value	RR	95% CI	p-value
Playgroup participation						
None	**1.7**	**1.2–2.2**	**< .001**	**2.0**	**1.4–2.9**	**< .001**
Either Wave 1 or 2	**1.8**	**1.3–2.4**	**< .001**	**2.0**	**1.4–2.8**	**< .001**
Both Wave 1 and 2	*Ref*	-	-	*Ref*	-	-
Attachment to friends^a^						
No attachment	**2.1**	**1.6–2.7**	**< .001**	**2.1**	**1.6–2.6**	**< .001**
Adequate attachment	*Ref*	-	-	*Ref*	-	-
Frequency of needing support and not getting it^a^						
Often/Very often	1.4	0.9–2.1	.102	1.2	0.8–1.7	.306
Sometimes	1.0	0.8–1.3	.833	1.0	0.7–1.3	.838
Rarely	*Ref*	-	-	*Ref*	-	-
Mother’s highest education^b^						
Year 10 or less	1.3	0.8–2.0	.273	1.4	0.9–2.2	.179
Year 11 or 12	1.4	1.0–1.9	.087	0.8	0.5–1.3	.461
Diploma or certificate	1.3	1.0–1.7	.055	1.2	0.9–1.5	.243
Bachelor degree or higher	*Ref*	-	-	*Ref*	-	-
Mother’s employment^b^						
Not employed	1.3	1.0–1.7	.059	1.3	0.9–1.7	.163
Employed, on maternity leave	**2.2**	**1.1–4.6**	**.030**	c	**-**	**-**
Part-time	*Ref*	-	-	*Ref*	-	-
Full-time	**1.5**	**1.1–2.0**	**.022**	1.1	0.8–1.5	.386
Equivalised household income tertile^b^						
Lowest	0.9	0.7–1.3	.694	1.2	0.8–1.7	.317
Middle	0.8	0.6–1.1	.113	0.9	0.7–1.2	0.529
Highest	*Ref*	-	-	*Ref*	-	-
Family Structure^b^						
Single parent	1.2	0.8–1.7	.436	0.8	0.6–1.2	.379
Other	*Ref*	-	-	*Ref*	-	-
Language spoken in the home^b^						
Non-English	1.4	1.0–2.0	.087	**1.8**	**1.3–2.5**	**.002**
English	*Ref*	-	-	*Ref*	-	-
Mother’s mental health^b^						
Within normal range	*Ref*	-	-	*Ref*	-	-
Elevated psychological distress	1.3	0.9–1.9	.129	0.9	0.6–1.5	.742

Note: Statistically significant relationships are written in bold (p < .05).

^a^ Measured at Wave 1.

^b^ Measures taken from the concurrent Wave (3 or 5).

^c^ Excluded for small cell size at Wave 5.

Having poor attachment to friends at Wave 1 (3–19 months), our proxy control for initial indicators of support from friends, was strongly associated with the risk of having no support from friends in later years, where these mothers were 2.1 times as likely as mothers with adequate friendship attachments to have no support from friends at both Wave 3 (4–5 years) and Wave 5 (8–9 years). The frequency of needing support at Wave 1 but not getting it was not associated with social support from friends at either Wave 3 or Wave 5.

Few of the socio-demographic characteristics considered were independently associated with the risk of not having support from friends in the multivariate models (Table 3). Mother’s employment status was associated with support outcomes when the study child was aged 4–5 years (Wave 3), but not at age 8–9 years (Wave 5). Mothers who worked full-time at Wave 3 were 1.5 times as likely to have no support from friends, and mothers employed on maternity leave 2.2 times as likely to have no support from friends compared with mothers who worked part-time. Mothers who did not speak English in the home were 1.8 times as likely as mothers who spoke English at home to have no support from friends at Wave 5 (8–9 years). In the multivariate models, frequency of needing support but not getting it, mother’s highest level of education, equivalised household income, family structure and mother’s mental health were not independently associated with support from friends at either Wave 3 or Wave 5.

## Discussion

This study examined the associations between playgroup attendance and the social support—or lack thereof—that mothers received from friends in later years. We found that persistent playgroup participation was a protective factor against having no support from friends in helping to raise the study child, both when children were aged 4–5 years (Wave 3) and four years later at 8–9 years (Wave 5). Compared to mothers whose child participated in playgroup at both Wave 1 (3–19 months) and Wave 2 (2–3 years), mothers whose child did not participate in playgroup, or who participated for only one wave, were almost twice as likely to report not receiving support from friends when their child was 4–5 years old (Wave 3), and twice as likely when their child was aged 8–9 years (Wave 5). These findings were independent of confounding variables including the mother’s initial attachment to friends and ability to obtain social support, education level, employment status, household income, family structure, language spoken at home and mental health status.

As this was an observational study, the observed associations between playgroup participation and receiving support from friends could be explained in a number of ways. One is that persistent playgroup participation leads to the development of friendship networks from which mothers can draw social support in later years. Participating in playgroups over a long-term period might also allow the opportunity to develop or reinforce the social confidence to engage with parents in other settings, for example, with the parents of their child’s school friends, which in turn may reinforce their ability to draw on social support from friends.

Another possible explanation is that mothers with a degree of social confidence and who are good at maintaining their friendships and networks are simply more likely to participate—and continue to participate—in playgroups. They may have chosen to attend a playgroup that already included friends or family members. Conversely, individuals who find it difficult to establish and maintain relationships, be that due to geography, circumstance or personality, may find it difficult to participate in social settings such as playgroups and to maintain social support resources over time. These mothers would therefore have less support from friends in the long term. However, our results remained after controlling for initial friendship attachments, suggesting that the relationship between playgroup participation and later friendship supports is unlikely to solely reflect the propensity of mothers to have friends or social supports and to participate in playgroups.

Even for mothers who are socially inclined to participate in playgroup, issues around equity of access may be a contributing factor. Access to playgroup is impacted by the availability of transport, appropriate venues, and most importantly, other families. In growth corridors or new residential areas, a lag in social infrastructure and family relocation can potentially result in the isolation of families with young children [33]. Disadvantaged families, previously shown to have lower rates of participation in playgroup [20], may also be excluded from certain playgroups where payment from participants is required to cover the cost of venue hire and other materials. Therefore some socially isolated mothers may have limited friendship support outcomes because playgroups are not accessible in some areas.

Our findings showed that the risks of having no support from friends were higher both for mothers who never participated in playgroup and those who participated at only one wave. Differences in outcomes only emerged for those mothers who persistently attended both at Wave 1 and 2, and even for this group of mothers 8% still reported having no support from friends in later years. It may be that some mothers attended several playgroups over the period, and did not establish friendships. Friendships, and the support that may stem from such friendships, are often not immediately established. Rather, it takes time for mutual trust, respect and affinity that stem from friendships to be established. As with any social network, there are likely to be a multitude of individual and group factors which will influence the level and types of social support that will stem from playgroup attendance [23]. Future research could examine whether parenting style and other factors such as personality contribute to the observed relationship between playgroup participation and social support outcomes.

In addition to the study child’s playgroup participation, mothers’ social support outcomes were also associated with a number of other socio-demographic characteristics in the unadjusted analyses. Mothers with elevated psychological distress were more likely to report not having support from friends compared with mothers without elevated levels of distress. This finding is consistent with previous literature demonstrating that poor social support is associated with an increased risk of maternal depression [34,35], and that mothers experiencing mental health problems are less likely to seek and/or recognise that they are in need of support [36,37]. Poorer social support outcomes were observed for mothers who worked full-time rather than part-time, and those with lower levels of education. Educational attainment, employment status and social support outcomes are typically associated in the wider literature [38]. The finding that single-parent families had poorer friendship support outcomes than two-parent families is also consistent with the wider literature. For example, Cairney et al. [39] found that, compared to coupled mothers, single mothers were more likely to report lower levels of perceived social support, social involvement and contact.

The literature concerning sources of social support for mothers of young children has previously examined a number of ways that mothers might connect with others and develop social support networks, including through organisations such as child care centres or preschool centres [17]. However, many Australian families either do not need, or cannot access, child care services or may have children who are too young for preschool, and therefore miss out on such opportunities. Additionally, by their nature, child care centres and preschools provide care and education services for children so that caregivers can attend to other commitments, such as work or study [19]. Playgroups, in contrast, actively promote the development of social support for the families who participate, where caregivers are expected to be actively involved in each session, interacting with both their child and with other caregivers. The active participation of caregivers also means that playgroups can address the social support needs of caregivers much earlier than these other sources, particularly for new parents experiencing a significant life transition with the arrival of a child. In the context of previous research therefore, our findings are not unexpected. However, research on the associated benefits of playgroup participation in particular, has been limited. As such, this paper contributes to the limited playgroup literature by demonstrating that persistent participation in playgroup is positively associated with friendship support outcomes.

Our findings also support the approach of playgroup organisations in promoting access to playgroups for disadvantaged and socially isolated mothers of young children. These results extend the findings of Hancock et al. [20] who found that children from disadvantaged families who attended playgroup had better social-emotional functioning and learning competence compared to children from disadvantaged families who did not attend playgroup. This study shows the value of playgroups for the development of parenting support networks, as well as the value of longitudinal data for examining such relationships.

This study has several limitations. As noted, the LSAC is an observational study, and hence we are unable to conclude whether there is a causal association between playgroup participation and social support outcomes. This study only used a broad level indicator of playgroup participation, thus we lacked information on the amount of playgroup attendance. In addition, there are many different types of playgroup, comprising different people (including both parents and facilitators) with different likes, dislikes, parenting styles and personalities. As such, playgroups are not a guaranteed source of social support, and it is possible that even mothers who persistently attend playgroup, be it the same or a variety of playgroups, will not establish friendships that extend beyond the playgroup setting. It is possible that, with more comprehensive data on frequency of attendance as well as type of playgroup attended, more informative results may have emerged.

Our indicator of social support from friends may also be somewhat limited, being a single-item measure. As such, some uncertainty may exist as to the extent to which the item captures social support from friends. However comparisons of how the item compared with related constructs, including friendship attachments, how often they saw their friends and how often they needed support but could not get it, suggested that the measure was a valid proxy.

In our study, we make the assumption that the mother would usually be the caregiver accompanying the child to playgroup. There is no Australian data available to determine which caregiver participates in playgroups with their child, but they can include mothers, fathers, grandparents and paid carers such as nannies. However, given that over 95% of the primary carers of the children in this study were mothers, we therefore assume that in most cases the mother attended playgroup with the study child. Had we been able to restrict analyses to cases where mothers accompanied the child to playgroup, it is possible that different associations may have been observed.

Despite these limitations, the findings provide evidence that playgroup participation is positively associated with friendship support outcomes for mothers. While social support can be provided from many sources, including partners, family, neighbours or work colleagues, mothers with extensive support networks may still find that playgroups help to widen and reinforce their support networks for times where other supports are unavailable. For mothers with limited social support options or those who are socially isolated, playgroups may be a helpful resource to establish friendships from which they can potentially draw social support.
